# A Review of the Biochemical Diagnostic Biomarkers in Migraine: New Perspectives in Diagnostics

**DOI:** 10.1155/prm/9478767

**Published:** 2025-12-08

**Authors:** Karolina Różycka, Natalia Siwak, Aleksandra Rucka, Joanna Bielewicz, Konrad Rejdak

**Affiliations:** ^1^ Department of Neurology, Medical University of Lublin, Lublin, Poland, umlub.pl

**Keywords:** biomarkers, CGRP, migraine, PACAP

## Abstract

**Background:**

The aim of this review was to evaluate studies concerned with substances which can be used as biochemical biomarkers of migraine (MH). Current MH diagnosis is based on the clinical criteria provided by International Headache Society (IHS) in the third edition of International Classification of Headache Disorders from 2018. Biochemical biomarkers could be useful in more precise and differential diagnosis which is important for proper choice of treatment.

**Methods:**

Preferred Reporting Items for Systematic Reviews and Meta‐analyses guidelines (PRISMA) were applied. MEDLINE (via PubMed), Web of Science, and Embase databases were searched in the recent 5 years. Studies exclusively focused on MH and biochemical biomarkers in adults and children, which can be applied as diagnostic tools, were conducted.

**Results:**

Finally, 31 researchers were assessed and discussed. Most of the presented studies were focused on calcitonin gene–related peptide (CGRP), pituitary adenylate cyclase‐activating peptide (PACAP), pentraxin 3 (PTX3), cytokines, and markers related to mitochondrial metabolism, making CGRP and PACAP possible diagnostic biomarkers. However, other agents have promising value.

**Conclusion:**

Based on the available studies, there are no specific substances which could be proposed as potential, practical, and useful biomarkers in HD. However, a few agents could be promising. The standardization of methodology would help in further investigations. Panels of biomarkers, easily obtained from blood but also from saliva and urine, could be interesting purposes for research in future.

## 1. Introduction

Migraine (MH) is a common disorder, affecting 18% of women and 6% of men, while chronic MH affects 2% of the global population, being an extremely disruptive disease, impacting people’s quality of life and ability to participate in work, family, and social events [1].

This is a neurological disorder characterized by attacks of recurrent headaches (at least 5). Currently, diagnosis of MH is established based on the clinical presentation. Criteria provided by International Headache Society (IHS) in the third edition of International Classification of Headache Disorders from 2018 are defining MH attacks regarding the time of lasting, its characteristics (localization, quality, intensity, aggravating factors) and the presence of accompanying symptoms, after exclusion of other probable diagnosis. Also clinical presentation directs the allocation of MH attacks into several groups depending on the presence of aura and frequency of headache episodes. We distinguish between episodic (EM) and chronic (CM) forms, with (MA) or without (MO) aura [2].

MH diagnosis needs very detailed appropriate history taken and exclusion of other MH‐like disorders, especially those manifesting with secondary headaches. The International Association for the Study of Pain [3] defines the pain as subjective experience. The lack of other clinical methods used during the evaluation of a patient with headache provides incorrect diagnosis and choice of ineffective treatments.

Some researchers indicate other, more‐expanded symptoms which are associated with MH than those described in Classification of IHS from 2018 [4] and postulate to revise it [5]. These observations confirm the inaccuracy of the MH diagnosis solely based on subjective assessment.

However, the clinical characteristics are still crucial to diagnosis and differentiate MH in the subtypes. Even if not perfect but can serve for development machine learning models which can predict treatment responses to commonly used MH preventive medications [6].

Anyways, access to more objective tools that can be useful and can support the clinical diagnosis is needed.

As defined by the National Institutes of Health (NIH), biomarkers are molecules that may be present in the blood or other tissues and organs. Their detection may indicate an ongoing pathological process or disease. Some biomarkers can act as predictive factors. The discovery of new biomarkers and the research conducted on them enable the development of diagnostics and treatment [7]. Research on the MH biomarkers has spanned diverse fields, including genetics, neuroimaging, and biochemical approaches.

For example, several studies have investigated correlations between MH attacks and structural changes seen in MRI [8]. Additionally, research is underway to predict treatment response based on radiological biomarkers [9].

Findings of the specific and sensitive biochemical biomarkers are the most desired because their easy and quick access. Application of such tests could be helpful in the process of the diagnosis of questionable cases and allocation to the particular subgroups. Unified diagnostic biomarkers for MH are needed to improve diagnostic accuracy and support more personalized treatment. Research has identified various molecules involved in different MH phases, both during attacks and between episodes.

However, there is still lack of agents, which could be practically useful for diagnostic purposes.

The aim of this review is to search and summarize up‐to‐date knowledge on the putative substances which can be used as exclusive diagnostic, biochemical biomarkers of MH to indicate those with promising potential in future.

## 2. Materials and Methods

This systematic review followed the Preferred Reporting Items for Systematic Reviews and Meta‐analyses (PRISMA) guidelines. Two independent and the headaches experienced neurologists conducted an electronic search in MEDLINE (via PubMed), Web of Science, and Embase databases. Keywords related to the topic were selected based on a literature using medical subjects headings (MeSH) descriptors for PubMed and Embase subject headings (EMTREE) for Embase. Then, the keywords were grouped into a single Boolean phrase as follows: (MH AND biomarkers). The review process, conducted according to PRISMA guidelines, involved analyzing the titles and abstracts to exclude studies that did not meet the inclusion criteria or met the exclusion criteria. Any discrepancies between the two reviewers were resolved through discussion or arbitration by a third reviewer. The articles were published within the last 5 years.

Four hundred eighty‐nine articles were identified. Papers without full text and an English version were not taken into any further considerations. Four hundred seventy‐nine publications were included in screening. Duplicate studies, letters to the editors, commentaries, erratum, books and documents, case reports, electronic supplementary, reviews and systemic reviews were excluded.

The remaining 101 publications were evaluated using inclusion and exclusion criteria as follows:

Inclusion Criteria: 1. Presence of the word “biomarkers” in the title or abstract, 2. Clinical studies on adults and children’s populations, 3. Studies focused solely on the diagnosis of MH.

Exclusion Criteria: 1. Experimental studies, in vitro studies, and animal studies, 2. Publications addressing biomarkers in the context of other aspects of MH, such as prognosis or preventive treatments, that do not pertain directly to MH diagnosis.

**Figure 1:**
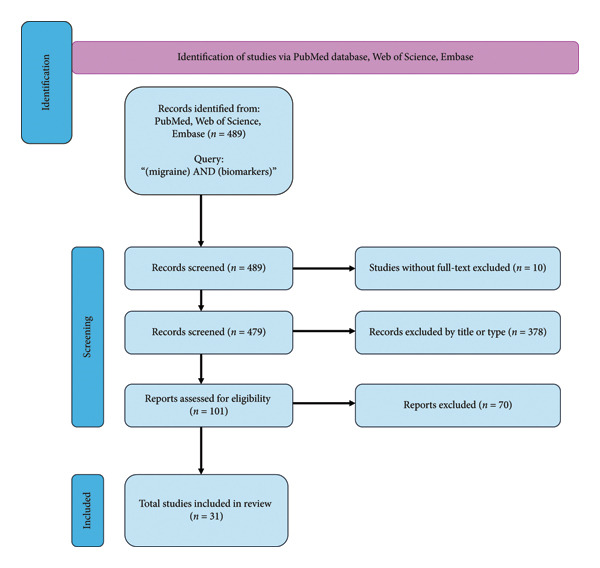
The flowchart for the selection process; n: number of studies.

Finally, 31 studies were chosen to be presented and discussed in this review (Figure 1).

The data extracted from each study were: (1) author and year, (2) number of participants, (3) type of MH (aura/without aura, episodic/chronic, during attack/between attacks), (4) methodology (materials), (5) results.

## 3. Results

Based on the above studies, several substances were being identified as a potential biomarker of MH. Although calcitonin gene–related peptide (CGRP), pituitary adenylate cyclase‐activating peptide (PACAP), pentraxin 3 (PTX‐3), cytokines, and markers related to mitochondrial metabolism (Fibroblast Growth Factor—21‐FGF‐21, Growth Differentiation Factor‐15—GDF‐15, and Hypoxia‐Inducible Factor‐1*α*—HIF‐1*α*) were discussed in most publications. Other agents were also studied and considered. Presented studies were examining diagnostic markers in adults and children.

### 3.1. Research Investigating CGRP

CGRP is a neuropeptide, consisting of 37 amino acids, belonging to the calcitonin family, which exists in two isoforms: *α* and β, encoded by two different genes, CALCA and CALCB, respectively. The *α* isoform is mainly found in the central and peripheral nervous system, while the *β* isoform is localized in the enteric nervous system [10]. Released during the trigeminovascular activation CGRP leads to neurogenic inflammation and acts as vasodilator. The first study conducted in 1990 by Goadsby et al. assessed plasma CGRP levels during spontaneous MH attacks in the external jugular vein but not in the cubital fossa. It showed that CGRP levels were elevated in migraineurs compared to healthy controls (HC) in both classic and common MH. However, the increase seen in classic MH was greater than that seen with common MH [11]. Involvement of CGRP in the MH pathophysiology indicated a new direction for treatment development.

A subsequent study by Vural et al. proved a higher serum CGRP concentration than that in HC during acute MH attacks. Interestingly, a positive correlation between serum CGRP level and pain intensity was also demonstrated. Serum CGRP did not differ across gender groups and presence of aura in subgroup analysis [12].

Alpuente et al. measured CGRP level in saliva at interictal, onset, post‐2 h of onset and end‐of‐attack in women with episodic MH. Salivary CGRP levels were interictally elevated and usually increased during an MH attack. Patients were classified into CGRP‐dependent (79.6%) and CGRP‐independent (20.4%) MH groups, based on changes in CGRP concentration between the pre‐ictal and paroxysmal phases. Salivary CGRP concentration was statistically significantly elevated during spontaneous MH attacks in the CGRP‐dependent MH group. Additionally, an association between the occurrence of photophobia and phonophobia in the CGRP‐dependent group was demonstrated [13].

Contrarily, Tchivileva et al. showed no significant changes between plasma CGRP concentrations in migraineurs measured interictally and HC [14].

Latif et al. measured CGRP and apolipoprotein E levels in blood obtained from antecubital vein. CGRP serum levels did not differ significantly among MH ictal, interictal phase, and HC. However, authors estimated that CGRP has a “fair” diagnostic accuracy to discriminate between MH ictal phase and healthy subjects [15].

Another study by Perez‐Pereda et al. has shown higher interictal serum CGRP in CM than both EM and HC, but its utility to discriminate MH categories was low [16]. There were no differences between EM and HC.

The purpose of the next studies was focused on the measurement of CGRP levels in different subgroups of migraineurs. Greco et al. found significantly higher levels in CM with the medications overuse than in EM patients [17].

CGRP was also investigated in patients with MH and patent foramen oval (PFO). A total of 153 patients with MA, 51 patients with PFO and 102 patients without were assessed. CGRP was assessed in serum at the onset of attack. Patients with MA and PFO had a higher level of CGRP, which could have a predictive value in this particular, clinical situation [18].

A study by Fan PC. et al. reported that the mean plasma CGRP levels in children with MH, both during attacks and interictal periods, were higher than those observed in non‐MH headache patients and HC. No notable association was found between CGRP levels and gender, EEG findings, or headache phase (whether during attacks or not) in individuals with MH [19].

A pediatric study conducted by Liu J. et al. demonstrated that CGRP levels in individuals with MH were elevated compared to the HC, both during attacks and interictal periods, irrespective of aura status. However, there were no significant differences in plasma levels of CGRP between the group MA and MO [20].

A recent pediatric study by Orak SA et al. reported that CGRP and vasoactive intestinal peptide (VIP) plasma levels were higher during the interictal period compared to the HC group. PACAP‐38 levels showed no significant differences in patients [21].

Discrepancies between the results of the above studies were observed. It may be due to the type of material used (plasma, serum, saliva), different collection methods, immunoassays used, different sampling time points, and phenotypic heterogeneity of patients.

There is still not enough data to use GCRP as the diagnostic marker of MH. However, it can be used as clinically relevant biomarkers for predicting the response to therapy, including anti‐CGRP MH drugs [22, 23].

The recent research investigating CGRP in MH is summarized in Table 1.

**Table 1:** The recent research investigating CGRP in MH.

Ref.	Year and author	Population	Comparison	Outcome
[18]	2024 Li C. et al.	153 participants	51 with migraine and PFO, 102 with migraine without PFO	CGRP was assessed in serum at the onset of attack. Patients with migraine and PFO had the higher level of CGRP.
[14]	2023 Tchivileva IE. et al.	80 participants	20 CG, 20 TMD without migraine, 20 migraines without TMD and 20 TMD with migraine	No significant changes were found between plasma CGRP concentrations in migraineurs measured interictally and HC
[12]	2022 Vural S. et al	135 participants	85 patients with acute migraine attacks (34 of the patients had MA, and 51 had MO) and 50 CG	Serum CGRP were higher in patients with migraine attack than the CG.
[13]	2021 Alpuente A. et al.	44 participants	22 women with EM and 22 CG	An increase in CGRP levels during migraine attacks was detected. CGRP levels in saliva were elevated interictally in CM and ictally in CGRP‐dependent.
[15]	2021 Latif R. et al.	28 participants	14 patients with migraine and 14 CG	Insignificant changes in serum CGRP levels within‐the‐group (migraine ictal vs. interictal). The comparison between the interictal phase in migraine patients and CG revealed insignificant differences in CGRP levels.
[16]	2020 Perez‐Pereda S. et al.	296 participants	101 patients with CM, 98 with EM and 97 CG	Serum CGRP levels were significantly higher in patients with CM compared to those with EM and the CG. There were no differences between EM and the CG.
[17]	2020 Greco R. et al.	55 participants	27 with EM and 28 with CM with the medications overuse	Found significantly higher levels CGRP in CM with the medications overuse than in EM patients.
[19]	2019 Fan PC. et al.	120 participants (children)	68 patients with MH, 30 patients with non‐MH and 22 CG, aged 5–18 years	CGRP levels in MH, during attacks and intricately, were higher than in non‐MH and CG.
[20]	2022 Liu J. et al.	153 participants (children)	76 with MH and 77 CG, aged 4–18 years	Serum CGRP levels were elevated in MH compared to CG, during attacks and interictally in patients MA and MO.
[21]	2025 Orak SA. et al.	79 participants (children)	39 with MO and 40 CG, aged 8–18 years	During the interictal period, CGRP and VIP plasma levels were higher than in CG.

CG, controls group; CGRP, calcitonin gene‐related peptide; CM, chronic migraine; EM, episodic migraine; MA, migraine with aura; MO, migraine without aura; PFO, patent foramen oval; SP, Substance P; TMD, chronic temporomandibular disorder; VIP, vasoactive intestinal peptide.

### 3.2. Research Investigating PACAP

PACAP is a neuropeptide found in two forms, PACAP‐27 and PACAP‐38. It is present in the brain and in peripheral organs such as the pancreas, gonads, respiratory, and genitourinary systems [24]. PACAP has vasodilatory effects that have been implicated in MH pathophysiology. Besides vasodilation, PACAP affects trigeminal nociceptor’s excitability by increasing accumulation of cAMP in trigeminal ganglia neurons [25]. PACAP‐38 pathway is considered as distinct from other MH provoking pathways such as CGRP. The first evidence of a role for PACAP‐38 in the pathophysiology of MH emerged in 2007. Birk et al. observed headaches inducing intravenous administration of PACAP‐38 [26]. Furthermore, PACAP levels elevated during MH attack decrease after sumatriptan application [27]. Clinical trials have shown the superiority of intravenous application of monoclonal antibody to PACAP versus placebo in reducing MH frequency [28].

Although PACAP’s role in MH pathophysiology is supported by many studies, there are only a few who tried to assess PACAP as a potential diagnostic biomarker.

Togha et al. observed higher serum PACAP‐38 levels intricately in EM patients compared to HC; however, the elevation of PACAP in the EM group was not significant compared with the CM group [29].

Contrarily, the study by Pérez‐Pereda et al. showed that serum PACAP‐38 levels were higher in patients with CM compared to patients with EM and HC, with no differences between EM and HC. PACAP‐38 compared to CGRP had more discriminative capability to distinguish CM from EM and HC [16].

Changes in PACAP concentrations in women with MH are influenced by their hormonal status which can explain the discrepancies in the results of the above studies [30].

Hanci F. and colleagues found that children with MH exhibited elevated plasma levels of PACAP‐38 and VIP compared to HC, whereas CGRP and SP concentrations did not differ significantly between the groups. PACAP‐38 and VIP levels were higher in MH patients than HC during both attacks and interictal periods [31].

A pediatric study by Liu J. et al. found elevated PACAP‐38 levels in MH patients compared to HC, regardless of attack phase or aura. No significant differences were observed in PACAP‐38 between MA and MO groups [20].

Recent researches investigating PACAP in MH are summarized in Table 2.

**Table 2:** Summary of recent research investigating PACAP in MH.

Ref.	Year and author	Population	Comparison	Outcome
[29]	2021 Togha M. et al.	89 participants	23 with EM, 36 with CM and 30 CG	Serum PACAP levels were significantly higher in the EM group than in the CG. There was no significant difference between the EM and CM groups, nor between the CM group and CG.
[16]	2020 Perez‐Pereda S. et al.	296 participants	101 patients with CM, 98 with EM and 97 CG	Serum PACAP levels were significantly higher in patients with CM compared to those with EM and CG. There were no differences between EM and CG.
[31]	2021 Hanci F. et al.	58 participants (children)	38 pediatric patients MO, aged 6–18 years and 20 CG	Plasma levels of PACAP‐38 and VIP were significantly higher in MO than in the CG, both during migraine attacks and interictal periods, whereas SP levels did not differ between the groups.
[20]	2022 Liu J. et al.	153 participants (children)	76 with migraine and 77 CG, aged 4–18 years	In migraine patients, PACAP‐38 levels were elevated compared to CG, both during attacks and interictal periods, as well as in MO and MA patients

CG, controls group; CM, chronic migraine; EM, episodic migraine; MO, migraine without aura; PACAP, pituitary adenylate cyclase‐activating peptide; SP, Substance P; VIP, vasoactive intestinal peptide.

### 3.3. Research Investigating PTX‐3

Pentraxin 3 is classified as an acute phase protein. PTX‐3, belonging to the pentraxin family, consists of a C‐terminal and a distinctive long N‐terminal domain. Unlike classical short pentraxins such as CRP, PTX‐3 features a unique, structurally unrelated N‐terminal region. It is synthesized by various cell types, including macrophages, endothelial, and vascular smooth muscle cells. Notably, PTX‐3 secretion is closely associated with vascular injury, indicating its specificity in response to vascular damage [32]. For this reason, PTX‐3 is mainly studied as an indicator of endothelial dysfunction [12]. PTX‐3 is also being investigated as a marker of endothelial dysfunction in MH.

Vural et al. demonstrated significantly higher PTX‐3 serum levels in patients with an acute MH attack than in HC. Serum PTX‐3 levels did not significantly differ between the MH subgroups with or without aura in their subgroups analysis [12]. The same study showed that there was no correlation between PTX‐3 levels and headache duration and severity.

Similarly, Gokdemir et al. showed the significant PTX3 serum increase in the attack of MA [33].

Dominiguez‐Vivero et al. studied PTX‐3 as a potential biomarker of endothelial dysfunction in CM. Diagnosis of CM was 68.4 times more likely in an individual with serum levels of PTX3 ≥ 832.5 pg/mL [34].

Recent researches investigating PTX‐3 in MH are summarized in Table 3.

**Table 3:** Summary of recent research investigating PTX‐3 in MH.

Ref.	Year and author	Population	Comparison	Outcome
[12]	2022 Vural S. et al.	135 participants	85 with acute migraine attacks (34 MA, 51 MO) and 50 CG	Serum PTX‐3 levels were higher in patients during a migraine attack compared to CG, but no significant differences were observed between MA and MO subgroups in the analysis.
[33]	2020 Gokdemir MT. et al.	88 participants	44 with acute migraine attacks and 44 CG	Elevated serum PTX‐3 levels were observed in patients during a migraine attack compared to CG.
[34]	2020 Dominiguez‐Vivero C. et al.	130 participants	102 CM patients and 28 CG	Diagnosis of CM was 68.4 times more likely in an individual with serum levels of PTX3 ≥ 832.5 pg/mL.

CM, chronic migraine; CG, controls group; MA, migraine with aura; MO, migraine without aura; PTX‐3, pentraxin 3.

### 3.4. Research Investigating Cytokines

Cytokines have a proven role in the regulation of pain. Proinflammatory cytokines such as tumor necrosis factor alpha (TNF‐α), interleukin 1β (IL‐1β), interleukin 6 (IL‐6), and anti‐inflammatory cytokines such as interleukin 10 (IL‐10) were also investigated in the pathogenesis of MH [35].

Han D. et al. have found that patients with MH had higher serum levels of IL‐6, IL‐1β, and TNF‐α compared to HC. The blood samples were obtained from jugular vein 2 h from the onset of attack. However, no significant differences could be demonstrated between MA and MO. Additionally, Han observed that CGRP levels correlated significantly with IL‐1 *β* and IL‐6, but not with IL‐2, IL‐10, and TNF‐α [36].

In the Thoga et al. research, serum levels of IL‐6 and TNF‐α were significantly higher in the group with CM compared to EM and HC, highlighting that as levels of inflammatory factors increased, headaches were more chronic in nature [37].

However, a recent study of Acarsoy et al. showed no association between blood‐based markers of the immune system and MH status [38].

Based on the different results of the above studies, the status of cytokines as a potential biomarker in MH is uncertain. Their levels can reflect the ongoing neuroinflammation in migraineurs patients. Dündar et al. found interictally increased serum levels of the molecules, which play a role in the inflammatory process, that is, visinin‐like protein 1 (Vilip‐1), YKL‐40, lipocalin‐2, and IL‐23 [39].

The recent researches investigating cytokines in MH are summarized in Table 4.

**Table 4:** Summary of recent research investigating cytokines in MH.

Ref.	Year and author	Population	Comparison	Outcome
[38]	2023 Acarsoy C. et al.	6,593 participants	995 with migraine, 5598 without migraine	Showed no association between blood‐based markers of the immune system and migraine status.
[37]	2020 Togha M. et al.	90 participants	71 patients with migraine (44 EM and 27 CM) and 19 CG	Serum levels of IL‐6 and TNF‐α were significantly higher among subjects with CM than the EM and CG.
[36]	2019 Han D. et al.	85 participants	47 patients with migraine (27 of the patients had MA, and 20 had MO) and 38 CG	The serum level of IL‐1β, IL‐6, TNF‐α, and CGRP in the migraine group were significantly higher than the normal group.

CG, controls group; CGRP, calcitonin gene‐related peptide; CM, chronic migraine; EM, episodic migraine; MA, migraine with aura; MO, migraine without aura IL‐interleukin; TNF‐tumor necrosis factor.

### 3.5. Research Investigating Markers Related to Mitochondrial Metabolism

Research indicates that mitochondrial dysfunction, energy metabolism disorders, and oxidative stress may be involved in the development of MH [40]. HIF‐1*α*, FGF‐21, and GDF‐15 are central to the development of these three conditions. HIF‐1*α* is a key transcription factor for cellular adaptation to hypoxia and mitochondrial regulation. Mitochondrial stress also influences FGF‐21 and GDF‐15 production, both biomarkers of mitochondrial diseases [41].

He J. et al. reported that MH patients had higher serum FGF‐21 and GDF‐15 levels than HC. Additionally, patients with CM were found to have higher levels of FGF‐21 compared to those with EM, whereas GDF‐15 levels did not differ significantly between the two groups [42].

Contrarily, Burow P. et al. found higher plasma GDF‐15 levels in the MH group than in HC, with no significant difference in FGF‐21 levels. There were no significant differences identified between EM and CM regarding levels of FGF‐21 and GDF‐15. Furthermore, the study demonstrated increased levels of FGF‐21 in patients experiencing greater pain intensity; however, this pattern was not observed for GDF‐15 [43].

Kilinc YB. et al. found that children with MH had significantly higher circulating levels of HIF‐1*α*, GDF‐15, FGF‐21, CGRP, and PACAP‐38 compared to HC. Peptide levels were higher in CM than EM and rose during attacks compared to asymptomatic periods. Serum HIF‐1*α* and FGF‐21 levels were positively correlated with frequency attacks [41].

The recent researches investigating markers related to mitochondrial metabolism in MH are summarized in Table 5.

**Table 5:** Summary of recent research investigating markers related to mitochondrial metabolism in MH.

Ref.	Year and author	Population	Comparison	Outcome
[42]	2023 He J. et al.	345 participants	221 patients with migraine (153EM, 68CM) and 124 CG	Serum levels of FGF‐21 and GDF‐15 were significantly higher in individuals with migraine compared to CG. In CM serum FGF‐21 levels, but not GDF‐15 levels, were significantly elevated compared to EM.
[43]	2021 Burow P. et al.	328 participants	230 patients with migraine and 98 CG	Plasma levels of GDF‐15 were elevated in the migraine group compared to the CG, while plasma FGF‐21 levels showed no significant difference.
[41]	2023 Kilinc YB. et al.	88 participants (children)	68 female pediatric MO and 20 female CG, aged 8–18 years	Serum levels of HIF‐1*α*, GDF‐15, FGF‐21, CGRP, and PACAP‐38 were significantly higher in migraine patients. The serum levels of these peptides were also higher in patients with CM than in EM, and higher in the ictal period than in the interictal period.

CG, controls group; CGRP, calcitonin gene‐related peptide; CM, chronic migraine; EM, episodic migraine; FGF‐21, fibroblast growth factor‐21; GDF‐15, growth differentiation factor‐15; HIF‐1*α*, hypoxia‐inducible factor‐1*α*; MO, migraine without aura; PACAP‐38, pituitary adenylate cyclase‐activating peptide.

### 3.6. Research Investigating Other Biomarkers: New Players in the Game?

Like CGRP and PACAP‐38, neurokinin A (NKA), a member of the tachykinin family, is another neuropeptide potentially engaged in MH pathophysiology. Tachykinins are neurotransmitters with potent excitatory effects in the central and peripheral nervous system. NKA probably contributes to the pathogenesis of MH by causing increased vascular permeability in response to trigeminal nerve activation. The meta‐analysis of Frederiksen et al. noted that NKA, but also serotonin (5‐HT), CGRP, endothelin‐1 (ET‐1), neuropeptide Y (NPY), PACAP‐38, and VIP blood levels were significantly elevated during MH attacks compared to the interictal phase [44].

Amylin is a 37‐amino‐acid peptide hormone that is closely related to CGRP. Because both peptides act on receptor AMY1, the overlap between both systems is observed [45]. However, in human research, differences are noted. Irmina et al. found interictal plasma amylin levels higher in CM than in EM and HC, while CGRP increased in CM when compared to HC, but not to EM [46].

All new possible MH biomarkers are still evaluated. The single studies investigating serum cystatin C (associated with MH without aura) [47], immunoglobulin IG1 [48] metabolites of tryptophan in urine, particularly 5‐hydroxyindoleacetic acid (5‐HIAA) (lower level interictally in EM) [49] were published.

Elevated inflammatory markers were also assessed in several studies. Their increase may indicate the severity of the disease [50, 51].

Another approach to identify the potential biomarkers of MH is the untargeted proteomics’ analysis to measure the complex nonspecific for MH proteins in serum [52] and CSF [53] or urine [54].

Some research has given negative results. Yazar et al. concluded that serum uric acid levels were not significant for MH subtypes [55]. Results of the study of Kim et al. do not indicate interictal plasma endothelin‐1 level as a marker for EM and CM [56].

Transient receptor potential Melastatin 3 (TRPM3) is a calcium‐permeable ion channel expressed in sensory neurons of the trigeminal ganglion and dorsal root ganglion, where it contributes to the transmission of neuroinflammatory pain signals. TRPM3 is coexpressed with MH‐related genes in the human trigeminal ganglion, and genetic variants of TRPM3 have been linked to an increased risk of MH. Ongoing studies are evaluating an orally administered TRPM3 antagonist as a potential acute treatment for MH attacks [57].

## 4. Discussion

Identifying MH‐specific biomarkers would be fundamental from a precision medicine perspective. They would allow accurate diagnosis and classification of subtypes. This is crucial for application of the proper, tailored therapy and further monitoring of treatment effects as for adults as for children. Attempts to define diagnostic biomarkers in MH have been ongoing for a long time. To date, no reliable biomarker candidates which can be useful in diagnosis have been identified in peripheral blood, cerebrospinal fluid, saliva, or urine [2, 58, 59].

Biomarker candidates are chosen from molecules involved in MH pathophysiology. While the exact mechanism of MH remains debated, several contributing factors have been identified [60, 61]. Nerves (particularly the trigeminal nerve), dural mast cells, and dural blood vessels interact via autocrine, paracrine, and endocrine mechanisms, which contribute to neurogenic inflammation associated with MH [62]. Neuropeptides including substance P, PACAP, acetylcholine (ACh), adenosine triphosphate (ATP), and CGRP, which are released from trigeminal sensory nerve endings, have the capacity to induce mast cell degranulation and provoke vasodilation within the dura mater. Mast cells then release mediators such as substance P, PACAP, histamine, nitric oxide, TNF‐α, and IL‐6, which further impact vascular and neural functions. Mediators that are released cause increased vascular permeability and blood flow, resulting in protein leakage, neurogenic inflammation, activation, and sensitization of meningeal nociceptors [62, 63]. At present, it remains undetermined whether mast cell activation precedes nociceptor activation, or if both mechanisms are triggered concurrently.

Our review found a small group of studies in the past five years focusing solely on the utility of biomarkers in diagnostics and adhering to PRISMA guidelines—26 in adults and 5 in children. Currently, there are no exclusive biomarkers identified for pediatric MH. These studies are challenging to compare due to varying methodologies, but their findings remain valuable and may inspire new directions in this area.

Most studies have examined vasoactive neuropeptides, particularly CGRP and PACAP, which rise during MH attacks. CGRP’s diagnostic value is well established in most studies, and its concentration changes are used as endpoints in both animal and human research. However, its receptors outside the central nervous system and resistance for anti‐CGRP treatments in some patient groups limit its diagnostic specificity. Additionally, CGRP increases with other clinical events, such as spontaneous and induced seizures. Nevertheless, CGRP remains the most promising diagnostic biomarker.

Numerous studies have demonstrated the pivotal role of CGRP in both the initiation and progression of MH attacks. These findings have facilitated the development of innovative therapies, including monoclonal antibodies targeting CGRP ligand or its receptors (anti‐CGRP MAbs), as well as small‐molecule CGRP receptor antagonists (Gepants) [64, 65]. A recent large European multicenter study has confirmed the effectiveness of anti‐CGRP MAbs for treating high‐frequency episodic and chronic MH [61]. However, some patients remain resistant to these therapies, highlighting the need for new treatment options.

The PACAP‐38 pathway is regarded as distinct from other MH‐inducing pathways, such as CGRP, highlighting its potential diagnostic value in addition to that of CGRP.

NKA or PTX‐3, engaged in the vascular function, is also the subject of research. There are still not enough studies on which NKA or PTX‐3 can be considered as potential biomarkers.

Cytokines, mainly proinflammatory ones, participate in neurogenic inflammation by being released from degranulated meningeal mast cells during MH attacks. Several studies [62, 66] have shown and discussed their role in MH pathogenesis, but their levels also rise in other inflammatory conditions. The controversial results of the above studies on cytokines are observed. However, elevated levels of cytokines such as IL‐6 and TNF‐α can be associated with CM, which may suggest the involvement of inflammatory processes in the MH chronification. Cytokines are not specific MH indicators and should only be considered as potential biomarkers in certain clinical situations.

Differences in results of the above‐mentioned studies indicate a need of proper methodology which should be based on comparable procedures [67, 68]. Different materials were used plasma, serum of venous blood obtained from differently localized veins, saliva, and urine. Also the different time points, ictal and intra‐ictal were chosen for materials collection. One should be also aware that the studied patients could use different medication for MH treatment. Since the studied substances are not specific for MH exclusively, they are involved in other physiologic and pathologic processes. The choice of an adequate control group without comorbidities is a challenge. A debate is needed whether and in which points further studies should be standardized. Easy access to obtain materials for biomarkers is important from the practical point of view. Measurement of biomarkers in saliva or urine in interictal phase seems an interesting idea for research in the future.

MH is heterogenic entity, so different patomechanisms in different subgroups should be responsible for clinical presentation. Furthermore, engagement and interplay of different, independent metabolic pathways can result in MH. This explains the presence of anti‐CGRP antibodies‐resistant populations. A panel of potential biomarkers rather than a single agent should be the aim of any further investigations.

The introduction of the machine learning models into the evaluation procedures of patients with headaches brings hopes for quicker and more precise diagnosis and the effective treatment choice [69]. The biochemical assessment could provide other valuable information used by artificial intelligence.

## 5. Conclusions

To date, no reliable biochemical biomarker candidates that can be useful in diagnosis have been identified. Current diagnoses depend on the 2018 International Classification of Headache Disorders’ clinical criteria. Further investigations should be continued because MH biomarkers would be helpful in more precise diagnosis with subtype classification which would allow us to choose a tailored treatment. Adequate agents could be used to monitor treatment and predict the MH chronification.

The biggest challenge is standardization of applied methodologies which are crucial for the analysis of data and enable a better comparison of study results.

Research of biochemical biomarkers can provide valuable information utilized by machine learning models.

## Conflicts of Interest

The authors declare no conflicts of interest.

## Funding

No funding was received for this manuscript.

## Data Availability

Data sharing is not applicable to this article as no datasets were generated or analyzed during the current study.
